# Special Issue: Development and Application of Statistical Methods for Analyzing Metabolomics Data

**DOI:** 10.3390/metabo11070451

**Published:** 2021-07-13

**Authors:** Jos Hageman, Jasper Engel

**Affiliations:** Biometris, Wageningen University and Research, Droevendaalsesteeg 1, 6708 PB Wageningen, The Netherlands; jasper.engel@wur.nl

In the last decade, the field of metabolomics has developed tremendously: it is now possible to routinely measure a wide range of metabolites for many specimens at reduced costs, opening the door to many exciting experiments. To match these developments, alternative statistical methods are required. This special issue was commissioned to offer a source of novel data analysis methods for metabolomics. Developments are reported in the whole range of the metabolomics pipeline, ranging from data preprocessing, “conventional” chemometrical analysis, and novel statistical procedures for outlier detection, network analysis, and fusion of omics data.

The paper by Seo Lin Nam [1] explores different data normalization strategies to improve urinary metabolomics analysis, and an improved procedure is proposed. Importantly, they demonstrate the impact of data processing on subsequent analysis. The risks of using default (normalization) approaches are highlighted.

Traditionally, techniques from chemometrics have been used for analysis of metabolomics data. Component models are often applied due to their ease of interpretation in score and loading plots. Importantly, Bevilacqua et al. show for PLS-DA that score plots may give misleading interpretations [2] and that these can be resolved by using cross-validated score plots. Yamamoto et al. show how more easily interpretable loadings are obtained in PCA by orthogonal smoothed PCA (OS-PCA) and propose a t-statistic for showing which metabolites contribute significantly to the PCA model loading [3]. Tinnevelt et al. focus on identification of significant metabolites in PLS-DA loadings and show that variable importance measures such as significance multivariate correlation offer better performance compared to penalized approaches such as sparse PLS-DA [4].

Component models make use of dimension reduction to deal with high-dimensional metabolomics data. Nowadays, many other regularization approaches are also employed in metabolomics for this purpose. In particular, the special issue highlights how shrinkage can be employed in metabolomics for improved effect size estimation and outlier detection. Brini et al. discuss the shrinkage of the matrix of the pairwise correlations between metabolites. They propose a shrinkage-based estimator for the Mahalanobis distance and demonstrate how this method may be used for outlier detection in one-class modeling [5]. Gillies et al. employ a multi-level Bayesian model for shrinkage of effect sizes while incorporating the uncertainty of the missing value imputation in the analyses [6]. They demonstrate by simulation that this approach more accurately estimates the effect sizes of significant metabolites. In addition, in case of missing data, the Bayesian model results in accurate imputation of its value.

Another approach to dealing with high-dimensional metabolomics data is to group related metabolites and test for significance of experimental factors at the group level. Such pathway analysis is explored by McLuskey et al. using a novel approach, PALS, which is based on pathway level analysis of gene expression data [7]. As an example, metabolites are grouped as metabolic pathways and by shared mass spectrometry fragmentation patterns. It is shown that PALS is more robust to missing features and noise compared to alternative methods. Similar to [1], it is highlighted that normalization can have a significant outcome on the analysis, and suggestions are made how PALS can be used to further investigate this.

An important group of techniques explores metabolite associations in networks. Jahagirdar et al. explore the use of correlation and mutual information (MI) to quantify association in 23 publicly available data sets and conclude that there is no significant benefit to using MI [8]. Iacovacci et al. focus in particular on association measures for short time series and show improved performance for Mahalanobis cosine and the hybrid Mahalanobis cosine in comparison to using Pearson’s correlation coefficient [9]. In a case study they demonstrate how the proposed measures can be used to encode multiple omics-specific levels of associations.

The topic of multi-omics fusion is also discussed in the review paper of Jendoubi [10]. They classify statistical multi-omics data integration approaches based on five criteria. Various aspects that lead to a particular choice for study design and data integration are discussed.

As guest editors we are grateful for the quality and wide range of work that was contributed to this special issue. All contributions combined, the articles in this special issue give a unique insight into the many currently ongoing developments of statistical analysis of metabolomics data. We look forward to the continued advancement of statistical methodology in the field that builds from the studies presented here.
